# Cell Model of Depression: Reduction of Cell Stress with Mirtazapine

**DOI:** 10.3390/ijms23094942

**Published:** 2022-04-29

**Authors:** Ana Salomé Correia, Sónia Fraga, João Paulo Teixeira, Nuno Vale

**Affiliations:** 1 OncoPharma Research Group, Center for Health Technology and Services Research (CINTESIS), Rua Dr. Plácido da Costa, 4200-450 Porto, Portugal; anncorr07@gmail.com; 2 Institute of Biomedical Sciences Abel Salazar (ICBAS), University of Porto, Rua de Jorge Viterbo Ferreira, 228, 4050-313 Porto, Portugal; 3 Department of Environmental Health, National Institute of Health Dr. Ricardo Jorge, 4000-053 Porto, Portugal; sonia.fraga@insa.min-saude.pt (S.F.); jpft12@gmail.com (J.P.T.); 4 EPIUnit-Instituto de Saúde Pública, University of Porto, 4050-600 Porto, Portugal; 5 Laboratório para a Investigação Integrativa e Translacional em Saúde Populacional (ITR), 4050-600 Porto, Portugal; 6 Department of Community Medicine, Information and Health Decision Sciences (MEDCIDS), Faculty of Medicine, University of Porto, Al. Prof. Hernâni Monteiro, 4200-319 Porto, Portugal; 7 Associate Laboratory RISE—Health Research Network, Faculty of Medicine, University of Porto, Al. Prof. Hernâni Monteiro, 4200-319 Porto, Portugal

**Keywords:** depression, SH-SY5Y cells, comet assay, mirtazapine, hydrogen peroxide, stress

## Abstract

Depression is a very prevalent and complex disease. This condition is associated with a high rate of relapse, making its treatment a challenge. Thus, an intensive investigation of this disease and its treatment is necessary. In this work, through cell viability assays (MTT and neutral red assays) and alkaline comet assays, we aimed to test the induction of stress in human SH-SY5Y cells through the application of hydrocortisone and hydrogen peroxide and to test the reversal or attenuation of this stress through the application of mirtazapine to the cells. Our results demonstrated that hydrogen peroxide, and not hydrocortisone, can induce cellular stress, as evidenced by DNA damage and a global cellular viability reduction, which were alleviated by the antidepressant mirtazapine. The establishment of a cellular model of depression through stress induction is important to study new possibilities of treatment of this disease using cell cultures.

## 1. Introduction

Major depressive disorder is a highly debilitating disease that is very prevalent throughout the world. This disease is characterized by symptoms that include anhedonia, sadness, lack of energy, difficulty in performing daily tasks and fluctuations in one’s weight and sleep cycle. In severe cases, this disorder may even culminate in death by suicide. It is a highly complex and heterogeneous disease in which several biological systems and molecular pathways are involved, making its study very complicated and challenging [1]. Indeed, one of the major problems associated with this pathology is the resistance to treatments and a high rate of relapse when treatments are discontinued, supporting the importance of an intensive and deep investigation of this disease and its therapeutic modalities [1,2].

It is important to establish methodologies for the study of this complex illness. As a behavioral condition, animal studies are crucial. However, a focus on the 3Rs policy of scientific investigation (replacement, reduction, and refinement) is increasingly important [3]. Thus, it is important to implement preliminary study strategies, such as research on cells, namely the neuronal and glial cell lines. Some examples are the rat clonal PC12 pheochromocytoma, human SH-SY5Y neuroblastoma, mouse HT-22 hippocampal, glioma C6 and BV2 microglial cell lines [4]. By using cell models, it is important to focus on biomarkers associated with depression. Thereby, it is possible to study these molecular mechanisms in detail at the cellular level. Theoretically, this type of study can be achieved, for example, with the use of hydrogen peroxide and glucocorticoids as stress inducers in cells, which is observed in vivo and has also been verified in some research studies [5,6,7,8,9,10,11].

In fact, the role of oxidative stress in depression has been studied and recognized. Individuals with depression typically have high levels of oxidative stress as well as low levels of antioxidant defenses, such as ascorbic acid and superoxide dismutase [12]. For example, a recent study on rats highlighted that the administration of N-acetylcysteine, an antioxidant, leads to an inhibition of neuronal injuries through its capacity to reduce oxidative stress, leading to antidepressant effects and supporting the involvement of oxidative stress in major depressive disorder [13]. Furthermore, it is important to mention that the presence of oxygen free radicals leads to the disease’s progression, contributing to the exacerbation of the effects mediated by pro-inflammatory pathways, culminating in an abnormality of brain functions and neuronal signaling [14]. Glucocorticoids (such as cortisol) are also known to be involved in the stress response through the hypothalamus-hypophysis–adrenal (HPA) axis [15]. Indeed, the presence of dysfunctions in this system and the presence of depression are correlated. Several studies demonstrate that chronic glucocorticoid exposure leads to disturbances in the HPA axis, which can lead to depression-like phenotypes [16]. Indeed, cortisol hypersecretion is considered a biological risk factor of depression [17].

**Figure 1:**
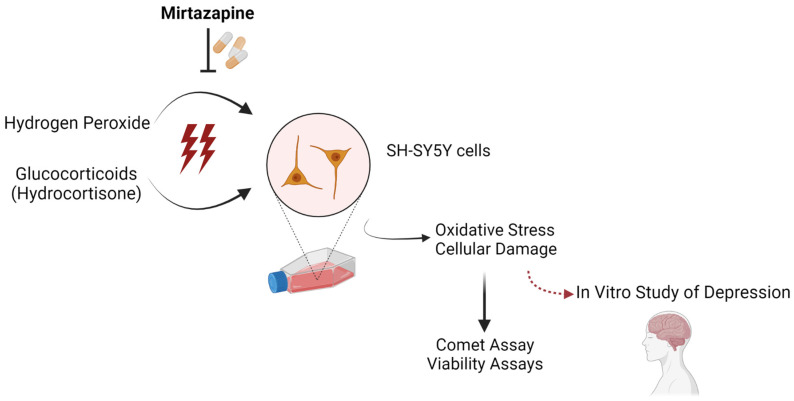
In this work, to establish an in vitro model of depression in human SH-SY5Y neuroblastoma cells, two stress inducers (hydrogen peroxide and glucocorticoids, particularly hydrocortisone) were used and antidepressant mirtazapine’s effectiveness against the induced oxidative stress was tested. Created with Biorender.com [18].

In this work, focusing on cell viability and DNA damage, we aimed to test the induction of stress in human SH-SY5Y neuroblastoma cells by exposing cells to glucocorticoids (hydrocortisone, the synthetic form of cortisol) and hydrogen peroxide (H_2_O_2_) as well as to test the reversal of this stress through the application of a clinically well-characterized antidepressant drug (mirtazapine) to the cells. Figure 1 illustrates the hypothesis in this work. Considering the obtained results, we proposed the study of depression using an in vitro model based on the oxidative stress that was effectively induced by using H_2_O_2_ and reverted by using mirtazapine. 

With this established model, the preliminary study of major depressive disorder becomes easier, making it possible to repurpose other drugs with potential in the treatment of this condition before the use of animals and more complex models of study.

## 2. Results

### 2.1. Effects of Hydrocortisone, Hydrogen Peroxide and Mirtazapine on Cellular Viability

**Figure 2:**
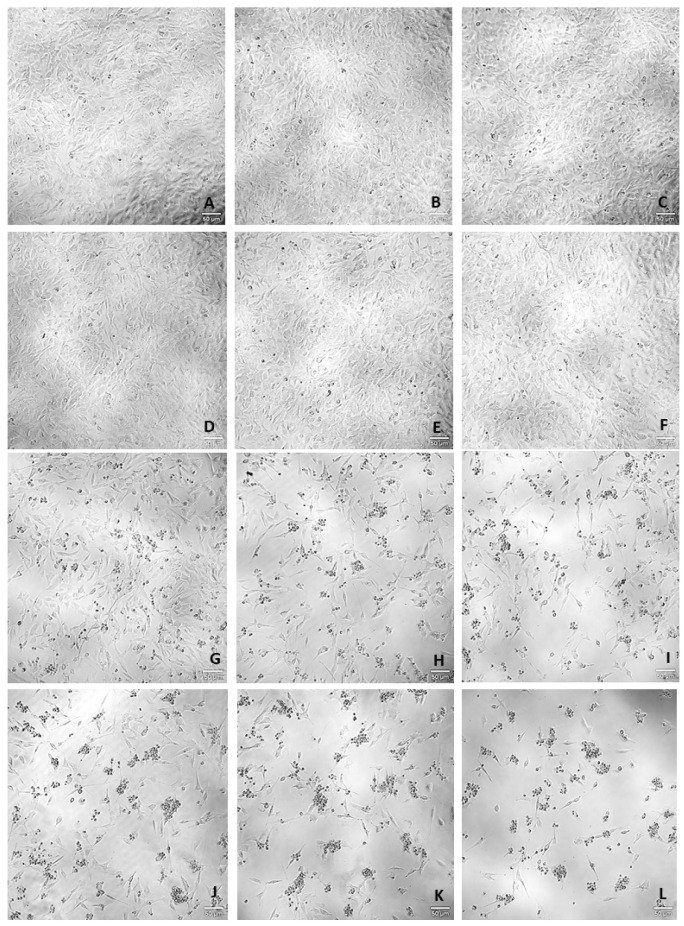
Representative images (100× total magnification) of SH-SY5Y cell morphologies after exposures to varied concentrations of hydrocortisone and H_2_O_2_. Cells were incubated with (**A**) vehicle (0.1% DMSO) (**B**) hydrocortisone 100 µM, (**C**) hydrocortisone 200 µM, (**D**) hydrocortisone 300 µM, (**E**) hydrocortisone 400 µM, (**F**) hydrocortisone 500 µM, (**G**) H_2_O_2_ 50 µM, (**H**) H_2_O_2_ 100 µM, (**I**) H_2_O_2_ 150 µM, (**J**) H_2_O_2_ 200 µM, (**K**) H_2_O_2_ 250 µM, and (**L**) H_2_O_2_ 300 µM. scale bar = 50 μm.

**Figure 3:**
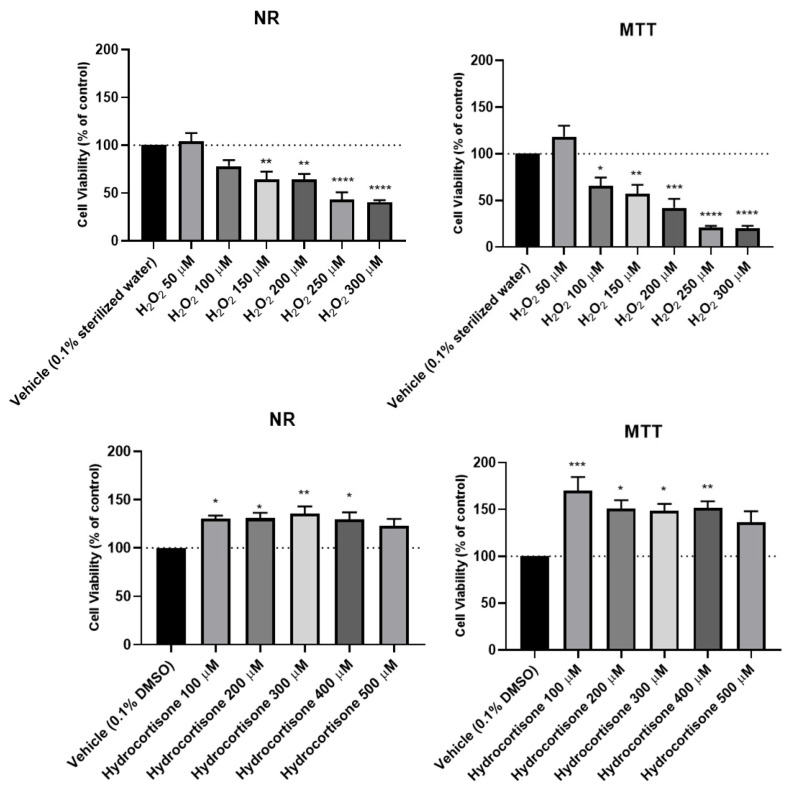
Effect of increasing concentrations of H_2_O_2_ and hydrocortisone on the viability of SH-SY5Y cells, for 48 h, obtained by the NR and MTT assays. The results are expressed as the percentage of each respective vehicle (0.1% DMSO for hydrocortisone and 0.1% sterilized water for H_2_O_2_) and represent the mean ± SEM of three independent cell culture preparations. Statistically significant * *p*< 0.05, ** *p* < 0.01, *** *p* < 0.001 and **** *p* < 0.0001 vs. vehicle.

**Figure 4:**
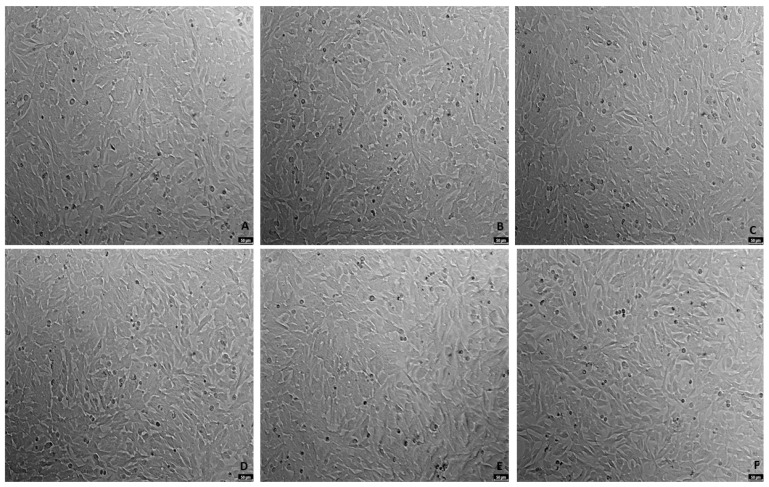
Representative images (100× total magnification) of SH-SY5Y cell morphologies after exposures to varied concentrations of mirtazapine. Cells were incubated with (**A**) vehicle (0.1% DMSO) (**B**) mirtazapine 0.01 µM, (**C**) mirtazapine 0.1 µM, (**D**) mirtazapine 1 µM, (**E**) mirtazapine 10 µM, and (**F**) mirtazapine 20 µM.

**Figure 5:**
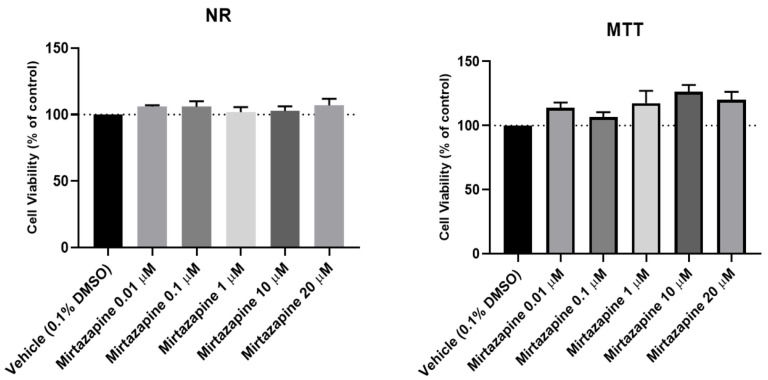
Effects of increasing concentrations of mirtazapine on the viabilities of SH-SY5Y cells for 48 h obtained by NR and MTT assays. The results are expressed as the percentage of vehicle and represent the mean ± SEM of three independent cell culture preparations.

To assess the effects of hydrocortisone, H_2_O_2_ (Figure 2 and Figure 3) and mirtazapine (Figure 4 and Figure 5) on the viability of SH-SY5Y cells, these compounds were added to the cells in increasing concentrations within a 48 h incubation period. After this period, morphological observations of the cells exposed with the different compounds under study were carried out. Then, the percentages of the viable cells were obtained using different cell viability methodologies: 3-(4,5-dimethylthiazol-2-yl)-2,5-diphenyl-2H-tetrazolium bromide (MTT) and Neutral Red (NR) assays, as described in the materials and methods section. For the H_2_O_2_, concentration-response curves (Figure S1) and half-maximal inhibitory concentration (IC_50_) values were also determined.

Our results revealed that hydrocortisone (Figure 2 and Figure 3), at any of the concentrations tested, did not lead to a decrease in cell viability, excluding its use as a stress-provoking agent in the proposed cellular model of stress. However, H_2_O_2_ (Figure 2 and Figure 3) significantly decreased cellular viability in a concentration-dependent manner, as observed in both cell viability methodologies. Analyzing the MTT concentration-response curve of this compound, an IC_50_ value of 132 µM was obtained. Additionally, analyzing the morphologies of the cells treated with this agent, it was clear that higher concentrations of H_2_O_2_ led to fewer cells as well as more damaged cells. Regarding the results obtained with the addition of mirtazapine (Figure 4 and Figure 5) to the cells, at any of the concentrations tested, this drug did not lead to a decrease in cell viability, consistent with the mechanism of action as an antidepressant, making this drug an ideal candidate for reversing the stress caused by the stress-provoking agent H_2_O_2_. Taken together, these results reveal that H_2_O_2_, and not hydrocortisone, is an ideal candidate for the proposed model of stress. Additionally, these results also show that mirtazapine does not lead to a decrease in cell viability and can be used as an antidepressant in the proposed model of stress.

### 2.2. Effects of the Combination of Mirtazapine with Hydrogen Peroxide in Cellular Viability

**Figure 6:**
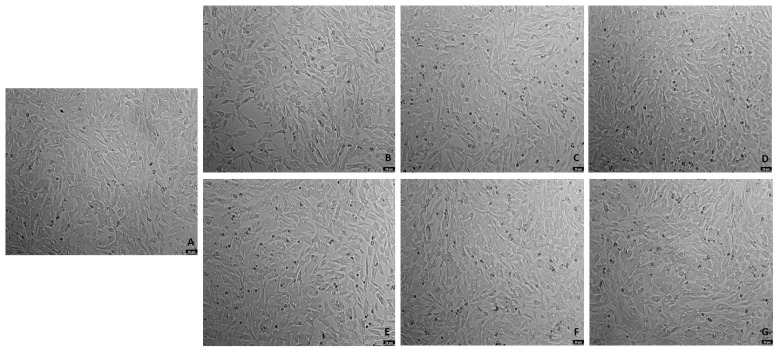
Representative images (100× total magnification) of SH-SY5Y cell morphologies after exposures to varied concentrations of the combination of mirtazapine with H_2_O_2_. Cells were incubated with (**A**) vehicle (0.1% DMSO) (**B**) H_2_O_2_ 132 µM, (**C**) H_2_O_2_ 132 µM + mirtazapine 0.01 µM, (**D**) H_2_O_2_ 132 µM + mirtazapine 0.1 µM, (**E**) H_2_O_2_ 132 µM + mirtazapine 1 µM (**F**) H_2_O_2_ 132 µM + mirtazapine 10 µM, and (**G**) H_2_O_2_ 132 µM+ mirtazapine 20 µM. scale bar = 50 μm.

**Figure 7:**
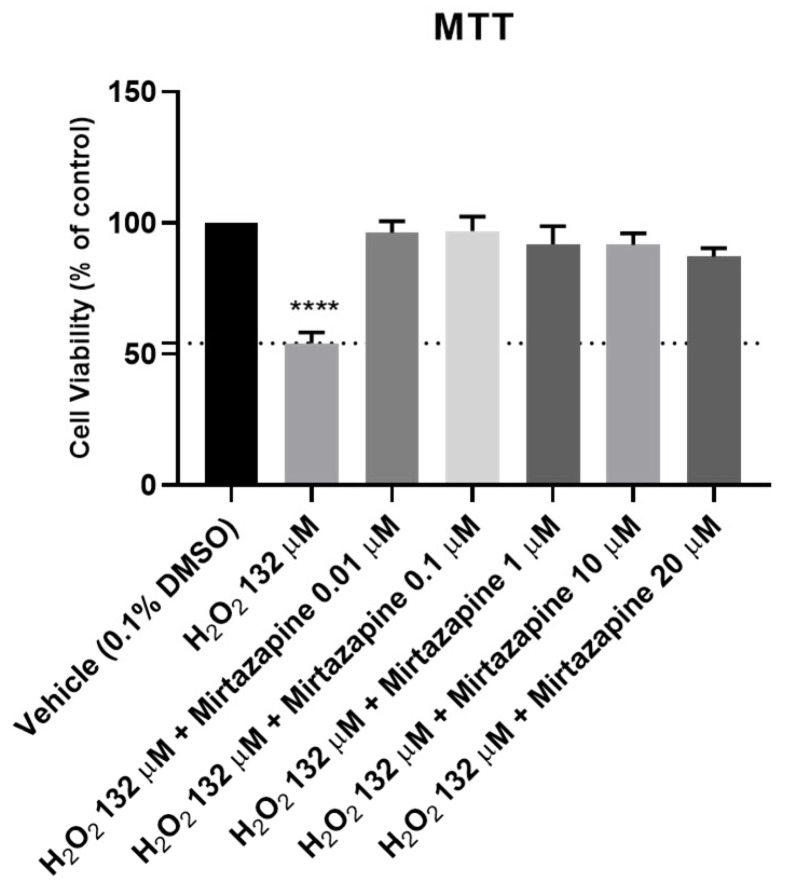
Effects of increasing concentrations of mirtazapine in combination with H_2_O_2_ on the viabilities of SH-SY5Y cells for 48 h obtained by using an MTT assay. The results are expressed as the percentage of vehicle (0.1% DMSO) and represent the mean ± SEM of three independent cell culture preparations. Statistical significance: **** *p* < 0.0001 vs. vehicle.

To evaluate the effects of mirtazapine in combination with H_2_O_2_ on the viability of SH-SY5Y cells, mirtazapine was added to the cells in crescent concentrations in combination with H_2_O_2,_ that was added to the cells in a concentration of 132 µM (representing the obtained IC_50_ value) for an incubation period of 48 h. After this period, morphological observations of the cells treated with this drug combination were carried out (Figure 6). Then, the percentages of viable cells were determined using the MTT assay (Figure 7), as described in the materials and methods section.

Analyzing the obtained results, it can be observed that mirtazapine, at any of the concentrations tested, was able to alleviate the decrease in the cell viability caused by H_2_O_2_ alone, leading to an increase in the cell viability to values similar to those obtained in the vehicle. Taken together, these results support the antidepressant activity of mirtazapine.

### 2.3. Effects of Hydrocortisone, Hydrogen Peroxide, Mirtazapine and the Combination of Mirtazapine with Hydrogen Peroxide on DNA Integrity

**Figure 8:**
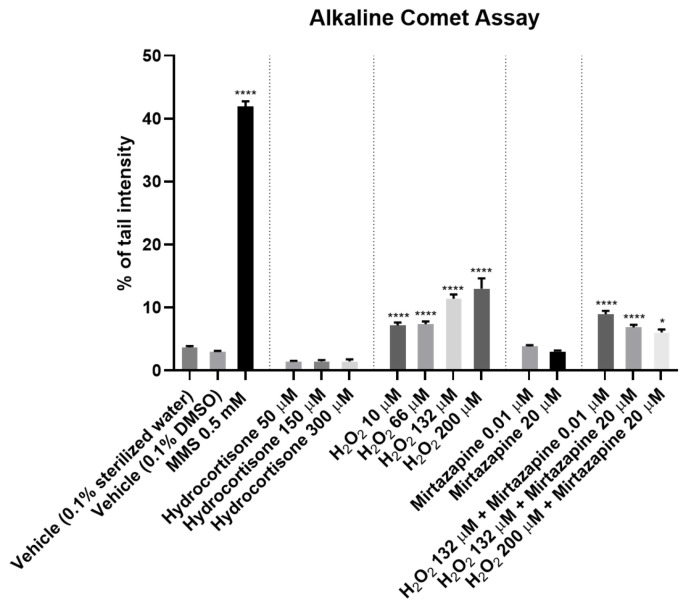
Effect of hydrocortisone, H_2_O_2_, mirtazapine and the combination of mirtazapine with H_2_O_2_ on the DNA integrity of SH-SY5Y cells for 48 h, as assessed by using alkaline comet assays. The results are expressed as the percentage of the tail intensity and represent the mean ± SEM of three independent cell culture preparations. Statistical significance: * *p* < 0.05 and **** *p* < 0.0001 vs. each respective vehicle.

**Figure 9:**
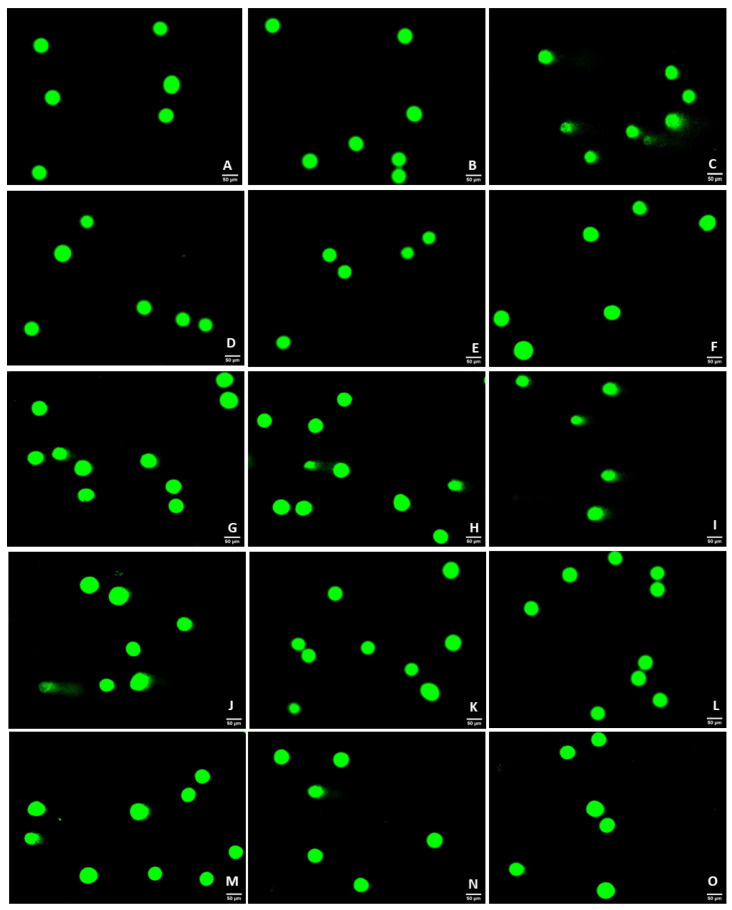
Representative images (200× total magnification) of SH-SY5Y cells after applications of increasing concentrations of hydrocortisone, H_2_O_2_, mirtazapine and the combination of mirtazapine with H_2_O_2_. These cells were stained with SYBR Gold, as described in the materials and methods section. Cells were treated with (**A**) 0.1% sterilized water vehicle, (**B**) 0.1% DMSO vehicle, (**C**) MMS 0.5 mM (positive control) (**D**) hydrocortisone 50 µM, (**E**) hydrocortisone 100 µM, (**F**) hydrocortisone 150 µM, (**G**) H_2_O_2_ 10 µM, (**H**) H_2_O_2_ 66 µM, (**I**) H_2_O_2_ 132 µM, (**J**) H_2_O_2_ 200 µM, (**K**) mirtazapine 0.01 µM (**L**) mirtazapine 20 µM, (**M**) H_2_O_2_ 132 µM + mirtazapine 0.01 µM, (**N**) H_2_O_2_ 132 µM + mirtazapine 20 µM, and (**O**) H_2_O_2_ 200 µM + mirtazapine 20 µM.

To assess the effect of hydrocortisone, H_2_O_2_, mirtazapine, and the combination of mirtazapine with H_2_O_2_ (Figure 8 and Figure 9) on the DNA integrity of the SH-SY5Y cells, these drugs were added to the cells in increasing concentrations for 48 h. After this period, the percentage of DNA damage (% tail intensity) of each cell was obtained using alkaline comet assays, as described in the materials and methods section.

Our results revealed that the H_2_O_2_ led to DNA damage in a concentration-dependent manner. Indeed, pronounced comet tails can be observed in the cells, representing more DNA damage (Figure 9G–J). The mirtazapine, as well as the hydrocortisone, did not lead to DNA damage, presenting similar values with the vehicle. Regarding the combination of mirtazapine with H_2_O_2_, these results confirm that mirtazapine was able to alleviate the DNA damage caused by increasing concentrations of H_2_O_2_. All these results support the cell viability studies, highlighting the role of mirtazapine as a drug able to reduce DNA damage caused by H_2_O_2_.

## 3. Discussion

**Figure 10:**
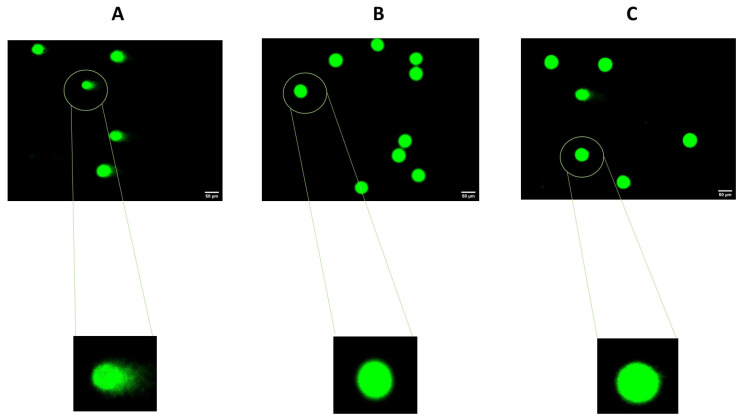
SH-SY5Y cells treated with (**A**) H_2_O_2_ (132 µM) presented more DNA damage than the cells treated with (**B**) mirtazapine (20 µM). This damage was evidenced by the comet tails, which reflect more DNA damage. When the (**C**) combination of mirtazapine (20 µM) and H_2_O_2_ (132 µM) was applied to the cells, the DNA damage was attenuated, which was confirmed with the presence of lower intensity of comet tails (compared to H_2_O_2_ alone).

Depression is a very prevalent and highly debilitating illness. Two of the major problems associated with this disease lie in a high rate of therapeutic failure and a strong difficulty in studying this condition from its molecular characterization to its treatment [1]. Thus, this work aimed to study depression using a cellular model to allow the study of this disease in a simpler, faster and more reproducible way with respect to the characteristics associated with the use of cell cultures in biomedical research. Therefore, we used well-characterized stress-provoking agents (cortisol and H_2_O_2_) related to the pathophysiology of depression [5,6,7,8,9,10,11]. Using these agents, we aimed to test the reversal/attenuation of this cellular stress through the application of antidepressant agents, such as mirtazapine, a known and well-characterized antidepressant [19]. Through cell viability methodologies (MTTs and neutral red assays) and alkaline comet assays, we aimed to characterize this model of cellular stress to make possible the use of other agents that may be promising in the treatment of depression. In summary, our results suggest that H_2_O_2_, not hydrocortisone, leads to a general decrease in cellular viability and DNA integrity, phenomena alleviated by the application of mirtazapine. These results were previously confirmed by cell viability techniques [11], but now they are also supported by the alkaline comet assays, highlighting the DNA damage present in the cells (Figure 10). The obtained results with the use of hydrocortisone may be explained by the ability of the cells to defend against the oxidative damage caused by cortisol in short periods. Furthermore, studies revealed that the increase in stress-induced cortisol is more pronounced at longer exposures, leading to increased levels of oxygen free radicals and DNA damage [20]. Thus, under our experimental conditions, cortisol exposure seems to not be enough to cause cell stress to the point of leading to significant damage to DNA (verified by using comet assays) and the cells’ viability (verified by using cell viability assays), even in high concentrations. Using H_2_O_2_, it was clear that this agent caused cell damage, compromising cell viability and the integrity of the cells’ DNA.

Regarding DNA integrity, previous studies have already reported increased levels of DNA strand-break cells after exposures to H_2_O_2_ [21,22,23,24]. However, the concentrations that were used were far higher (up to 1 mM) than the concentration tested in our study (132 μM). Additionally, most of the studies reported short incubation periods for H_2_O_2_. Nevertheless, we chose a 48 h period because we could observe if there was any cellular recovery from the damage caused by H_2_O_2_ in a longer period than most of the studies in that regard. This compound leads to an increase in oxidative stress, leading to an increase in free oxygen radicals, a phenomenon that is implicated in depression [13]. Thus, the application of H_2_O_2_ induces oxidative stress, similar to what is observed in individuals with this condition. In turn, mirtazapine leads to a protective effect on cells against this oxidative stress, highlighting the role of oxidative stress in depression and enabling the study of new therapeutic agents in its attenuation and reversal. Possibly, mirtazapine, by acting on serotonergic receptors [19], induces neuroprotection mechanisms that manage to oppose the harmful effects of H_2_O_2_. Furthermore, it is known that this antidepressant drug acts on the gene expressions of pro-apoptotic (Bax and p53) proteins, reducing their expressions [11]. With the application of this drug, there is also evidence of reduced neurite atrophy, a phenomenon that is evidenced in depression [25,26,27]. By establishing a stress model that is similar to the oxidative stress that occurs in individuals with depression, the doors were opened to the studying of other compounds that may be able to reverse or attenuate this damage. This study allows us to broaden the screening of drugs that can be repurposed in the context of depression, allowing the preliminary study of this disease to be more simplified, faster, and reproducible, focusing on individual molecular mechanisms. However, it is always important to keep in mind that depression is a highly complex disease in which several molecular/cellular mechanisms are involved. Several molecular biology studies as well as animal studies are crucial in the context of this disease.

## 4. Materials and Methods

### 4.1. Materials

Dulbecco’s Modified Eagle’s Medium (DMEM), Fetal Bovine Serum (FBS) and penicillin-streptomycin mixture were obtained from Millipore Sigma (Merck KGaA, Darmstadt, Germany). Thiazolyl blue tetrazolium bromide (MTT; cat. no. M5655), neutral red solution (cat. no. N2889), mirtazapine (cat. no. M0443), hydrocortisone (cat. no. H0888), hydrogen peroxide (30%; Perhydrol™; cat. no. 1.07209), Methyl Methanesulfonate (MMS; cat. no. 129925) and low melting point (LMP) agarose were purchased from Sigma-Aldrich (Merck KGaA, Darmstadt, Germany). SYBR^®^ Gold solution was obtained from Invitrogen (Waltham, MA, USA). Normal Melting Point (NMP; SeaKem LE agarose) agarose was supplied by Lonza (Basel, Switzerland). Formamidopyrimidine-DNA Glycosylase (FPG) enzyme was obtained from New England Biolabs (Ipswich, MA, USA).

### 4.2. Cell Culture

Human SH-SY5Y neuroblastoma cells (American Type Culture Collection, Manassas VA, USA) were maintained at 37 °C in 95% air and 5% CO_2_. These cells grew in DMEM (10% of FBS and 1% of a mixture of penicillin (1000 U/mL)/streptomycin (10 mg/mL)). SH-SY5Y cells are adherent cells; they were cultured in a monolayer and subcultured when the cells reached a confluence of 75–80%. Before each experiment, trypsin was added to the cells (0.25% trypsin-EDTA). Next, cells were centrifuged (1100 rpm for 5 min; Hettich, Tuttlingen, Germany) and seeded at a density of 4.2 × 10^4^ cells/cm^2^ in 96-well plates for the viability assays or in 48-well plates for the comet assays. Cells were used with a maximum passage number of 15.

### 4.3. Cell Treatment

Mirtazapine and hydrocortisone were dissolved in DMSO (0.1% in cell culture medium). The concentrations tested in the cells ranged between 0.01 µM–20 µM and 50 µM–500 µM, respectively. H_2_O_2_ (10 µM–300 µM) was dissolved in sterilized water (0.1% in cell culture medium). A stock solution of MMS (11.795 M) was prepared in sterilized water and further diluted in cell culture medium to a working concentration of 0.5 mM. For mirtazapine, hydrocortisone and mirtazapine/H_2_O_2_ combinations, vehicles were composed of 0.1% DMSO in cell culture medium. For H_2_O_2_, vehicle was composed of 0.1% of sterilized water in cell culture medium. All the treatments were tested in a period of 48 h after the cell attachment to the plates except MMS, which was used as a positive control for comet assays, being in contact with the cells for 1 h. For all the combinations tested, both agents were added simultaneously.

### 4.4. Cell Morphology Assessments

Cell morphologies were assessed using the Leica DMI6000 B Automated Microscope (Wetzlar, Germany) to observe and capture images of SH-SY5Y cells after all the treatment conditions.

### 4.5. Cell Viability Assays

After the 48 h cell treatments, cellular viabilities were evaluated by using MTT and NR assays. For the MTT assays, following the removal of the culture medium, 100 µL of MTT (0.5 mg/mL in PBS) was added to each well. Then, protected from the light, the cells were incubated for 3 h. Next, MTT was removed and 100 µL of DMSO was added to each well. Finally, absorbance values (570 nm) were obtained in the automated microplate reader (Tecan Infinite M200, Zurich Switzerland). For the NR assay, following the removal of the culture medium, 100 µL of NR medium (1:100 in culture medium) was added to each well. Then, the cells were incubated for a period of 3 h (protected from the light). After that, the cells were washed in PBS (150 µL), and NR destain solution (50% of 96% ethanol, 49% deionized water and 1% glacial acetic acid; 150 µL per well) was added to the cells. Next, absorbance at 540 nm was obtained in the automated microplate reader.

### 4.6. Alkaline Comet Assays

DNA damage after exposure to the tested agents was assessed by using the alkaline comet assays, as previously described [28]. Cells exposed to MMS (0.5 mM, 1 h) served as positive controls. Briefly, after their exposures, cells were washed with pH 7.4 PBS (without calcium/magnesium), detached by using trypsinization (150 µL/well for 5 min) and suspended in pH 7.4 PBS (without calcium/magnesium). Cells were then centrifuged (300× *g* for 5 min), the supernatant was discarded and the pellets were resuspended in ice-cold pH 7.4 PBS (without calcium/magnesium). Cells were counted in a Neubauer chamber and 6.0 × 10^3^ cells were transferred to a microcentrifuge tube, centrifuged at 400× *g* for 5 min and then embedded in 100 μL of 0.6% LMP agarose. Next, 5 μL of each sample was placed on the slides precoated with 1% NMP agarose using a high-throughput system of 12-minigel comet assay unit (Severn Biotech Ltd.^®^, Kidderminster, UK). Then, after agarose polymerization (4 °C for 10 min), the slides were incubated at 4 °C for 1 h, protected from light in lysis solution (2.5 M of NaCl, 100 mM of Na_2_EDTA, 10 mM of Tris-base, 250 mM of NaOH at pH of 10 and 1% Triton-X 100). Next, the slides were washed with cold H_2_O (4 °C for 3 × 5 min) and then immersed in electrophoresis buffer (1 mM of Na_2_EDTA and 300 mM of NaOH at pH of 13) for 30 min at 4 °C in the electrophoresis platform for DNA unwinding. Then, the electrophoresis ran for 20 min at constant 30 V (0.9 v/cm). At the end of the electrophoresis, the slides were washed with PBS (pH of 7.4; 2 × 5 min) and deionized H_2_O (1 × 10 min), followed by fixations in 70% and 96% ethanol (5 min each). Then, the slides were dried overnight and protected from light at room temperature. Finally, all the slides were stained with a 1:10,000 dilution of SYBR^®^ Gold in TE buffer (Tris-HCl (10 mM) and EDTA (1 mM) at pH of 7.5–8) for 20 min, observed in the Motic BA410 ELITE series microscope, equipped with a complete EPI fluorescence kit, and analyzed using the image analysis software Comet Assay IV (Perceptive Instruments, Staffordshire, UK). The DNA percentages in the comet tails (% tail intensity) were obtained for 100 cells per experimental condition.

### 4.7. Statistical and Data Analyses

The obtained results were represented as mean ± SEM of three independent cell culture preparations. Statistical analyses between control and treatment conditions were performed with Student’s *t*-test and one-way ANOVA tests. The differences were considered statistically significant when *p* value was <0.05. All the statistical analyses, constructions of graphs and calculations of IC_50_ values were performed using software GraphPad Prism 8 (San Diego, CA, USA).

## Supplementary Materials

The following supporting information can be downloaded at: https://www.mdpi.com/article/10.3390/ijms23094942/s1.
