# Supplementary material for: Cell Model of Depression: Reduction of Cell Stress with Mirtazapine
Source: Int J Mol Sci. 2022 Apr 29;23(9):4942. doi: 10.3390/ijms23094942 (PMC9099543; doi:10.3390/ijms23094942)
Supplement: Supplementary file 1 [file ijms-23-04942-s001.zip › ijms-1610565-supplementary.pdf]

International Journal of *Molecular Sciences*

MDPI

# Supplementary Materials

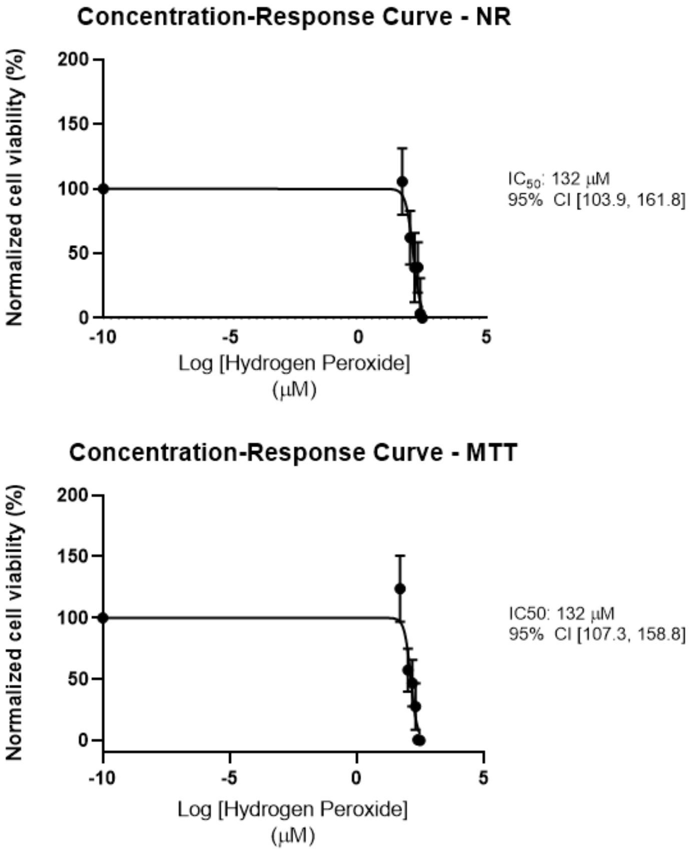


**Figure S1.** Concentration-response curves and $IC_{50}$ values (with 95% confidence intervals (CI)) for increasing concentrations of hydrogen peroxide on the viability of SH-SY5Y cells, for 48h, obtained by NR and MTT assays. The results are expressed as the percentage of each respective vehicle and represent the mean ± SEM of three independent cell culture preparations.
